# Enhancing Public Health Surveillance for Influenza Virus by Incorporating Newly Available Rapid Diagnostic Tests

**DOI:** 10.3201/eid0801.010067

**Published:** 2002-01

**Authors:** Paul V. Effler, Man-Cheng Ieong, Tammy Tom, Michele Nakata

**Affiliations:** Hawaii State Department of Health, Honolulu, Hawaii, USA

**Keywords:** influenza, surveillance, laboratory techniques, procedures

## Abstract

Beginning with the 1999-2000 influenza season, physicians throughout Hawaii ordering a viral culture for patients with suspected influenza were also offered influenza rapid testing. We compared the number of viral respiratory cultures sent to the Hawaii Department of Health and the number of providers who participated in influenza surveillance over consecutive influenza seasons. The number of viral respiratory cultures rose from 396 to 2,169 between the 1998-1999 and 2000-2001 influenza seasons, and the number of providers submitting >1 influenza culture increased from 34 to 327, respectively. The number of influenza isolates obtained each season also increased (from 64 to 491). The available data suggest that the changes observed in Hawaii’s influenza surveillance were not secondary to differences in influenza activity between seasons. This is the first evaluation of integrating influenza rapid testing into public health surveillance. Coupling rapid tests with cultures appears to be an effective means of improving influenza surveillance.

“The importance of surveillance to the detection and control of emerging microbial threats cannot be overemphasized.”

Institute of Medicine (*1*)

The recent, highly fatal, outbreak of human illness caused by a novel influenza A virus H5N1 in Hong Kong has dramatically underscored the importance of monitoring influenza activity in the United States as a part of national preparedness for a pandemic (*2*,*3*). Ongoing, comprehensive surveillance is vital to addressing influenza because influenza A viruses have the capacity to undergo abrupt shifts in the major antigenic determinants of their surface proteins (*4*); these shifts can give rise to novel influenza viruses capable of producing catastrophic pandemics (*5*). In 1918 an estimated 20 million people died from a new influenza A H1N1 virus strain (*6*). Milder influenza pandemics in 1957 and 1968 killed an estimated 90,000 people in the United States (*1*).

Enhancing our capacity to detect novel viruses with pandemic potential requires establishing comprehensive programs to culture and subtype influenza isolates in circulation during outbreaks and annual epidemics, in other words, virologic surveillance. With this goal in mind, the Hawaii State Department of Health (HDOH) sent letters in the fall of 1998 to all licensed physicians in the state encouraging them to collect specimens for viral culture when evaluating patients suspected of having influenza. Although these cultures were offered at no cost to the physician or patient, little increase in specimen submissions was observed during the 1998-99 influenza season when compared to prior years. Subsequent discussions with providers indicated that there was little incentive to collect viral culture specimens because culture results typically take 2 to 8 weeks to be finalized and thus are not perceived as useful for clinical management.

A more timely diagnosis of influenza is possible with rapid antigen tests (*7*). The advent of rapid testing for influenza has raised concern among public health professionals, however, because of the potential of such tests to undermine virologic surveillance. Some authorities have predicted that submissions of specimens for viral isolation would decrease as rapid antigen test kits are improved and become more widely available (*3*). The rapid tests currently available cannot distinguish novel virus subtypes from subtypes already known to be in circulation. If fewer viral isolates are obtained as a consequence of adopting point-of-care rapid tests, the result may be a reduction of our capacity for early detection of the next influenza pandemic.

We present an evaluation of the impact of incorporating rapid influenza testing into ongoing virologic surveillance activities.

## Methods

### Standard Virologic Surveillance

The virologic influenza surveillance system in Hawaii before the 1999-2000 influenza season consisted of physicians submitting cultures for influenza directly to HDOH (direct submissions). HDOH supplied the specimen collection materials, transported specimens, and processed the specimens without charge. There were no restrictions on the number of specimens that could be submitted. This service had been available for many years but was actively promoted through a mail-out in 1998 addressed to physicians throughout the state. Commercial clinical laboratories in Hawaii do not perform influenza viral cultures locally, and very few specimens are forwarded from these laboratories to reference laboratories on the U.S. mainland (the number of influenza cultures sent out of state has not exceeded 25 specimens for any of the major laboratories during each of the last four influenza seasons [F. Liu, X. Zheng, pers. comm.])

### Enhanced Virologic Influenza Surveillance for 1999-2000 and 2000-01

Beginning with the 1999-2000 influenza season, and again during the 2000-01 season, all licensed physicians in the state were informed by mail that rapid influenza tests were available to clinicians who also wished to order an influenza culture for their patient. The letter recommended considering the diagnosis of influenza in patients with a fever ≥37.8°C accompanied by a cough or sore throat.

HDOH supplied viral specimen collection kits and rapid test kits to four private clinical laboratories that provide service to approximately 80% of the state’s population, at no charge to the laboratory (*8*). The specimen collection kits contained two sterile dacron swabs, one sterile polystyrene tube, one tube containing viral transport medium (VTM Micro Test, Inc.; Lilburn, GA), and instructions for collection and submission of the appropriate specimens. Participating commercial laboratories were responsible for distributing these supplies to their satellite specimen collection locations (>70 distinct locations overall). To obtain both a rapid influenza test and viral culture, two throat or nasopharyngeal swabs were required, one placed into the sterile polystyrene tube and the other into the tube containing VTM. Specimens were transported through usual specimen transport channels.

Rapid influenza tests were performed at participating commercial laboratories, and the results were reported to the submitting provider and HDOH. Laboratories were not allowed to bill for the cost of rapid test kits provided by HDOH but could charge for performing the test per their usual practices. The specimen in VTM was then forwarded from the commercial laboratory to the Hawaii Public Health Virology Laboratory (HPHVL, a World Health Organization [WHO] collaborating virology laboratory) for culture, typing, subtyping, and antigenic characterization.

HDOH purchased the influenza rapid tests through a competitive bid process. The primary criteria used for selecting the BioStar FlU OIA test (Boulder, CO) was cost (<U.S.$5 for the first 1,000 tests and <$10 for any additional tests). The test manufacturer provided training on performing the rapid test to HPHVL and commercial laboratory staff.

For this report, the number and results of influenza cultures submitted and the number of distinct providers who submitted at least one viral culture during the 1997-98, 1998-99, 1999-2000, and 2000-01 influenza seasons were determined. The influenza season was defined as the period beginning with week 40 of the first year extending through week 20 of the next year, that is, early October through mid-May. In addition, for the 2000-01 influenza season, we calculated the positive and negative predictive values of the FlU OIA Rapid Test as compared to influenza viral culture for all specimens with complete test results as of May 21, 2001.

### Comparison Data

After observing a change in the number of viral cultures submitted between the 1999-2000 influenza season and the previous season, we conducted an assessment to determine if this difference might be explained by factors other than incorporation of rapid tests into the surveillance effort. Data regarding the number of influenza cultures performed at sites other than Hawaii for the 1997-98, 1998-99, and 1999-2000 influenza seasons were obtained from the Centers for Disease Control and Prevention (CDC) for approximately 75 other WHO collaborating virology laboratories located throughout the United States (*9*). Most of the participating laboratories are located in state or local health departments with a smaller number in universities or hospitals. During the 1999-2000 influenza season, 44 of the WHO laboratories (excluding Hawaii HPHVL) reported information for at least 25 weeks of the season; 37 (84%) of the 44 laboratories had also supplied reports for at least 25 weeks during each of the two prior influenza seasons, and these laboratories were included in our analysis.

Data from physician offices in Hawaii participating in the U.S. Influenza Sentinel Physician Surveillance Network (ISPSN) were used as a measure of the relative severity of Hawaii’s past four influenza seasons (*10*,*11*). ISPSN sites report the total number of patient visits and the number of visits for influenza-like illness on a weekly basis during the influenza season. Influenza-like illness was defined as a fever of ≥37.8°C accompanied by a cough or sore throat. Weeks for which the proportion of visits for influenza-like illness exceeded 3% were defined as having increased influenza activity (*10*).

## Results

The total number of specimens submitted to HPHVL for influenza culture increased sharply during the first season that rapid tests were introduced into influenza surveillance; this trend continued into the 2000-01 season (Table 1). The number of influenza isolates obtained tripled between the 1998-99 and 1999-2000 influenza seasons and then tripled again between the 1999-2000 and 2000-2001 seasons. A nearly 10-fold increase in the number of distinct providers who submitted influenza cultures was observed between the 1998-99 and 2000-01 influenza seasons.

**Table 1:** Number of influenza cultures submitted, isolates obtained, sub-typed and antigenically characterized, and the number of providers participating in surveillance, compiled by influenza season, Hawaii

	

	Influenza season^ a^			
Number of:	1997-1998	1998-1999	1999-2000^b^	2000-2001^b^
Cultures submitted	306	396	1112	2169
Influenza isolates	73	44	137	491
Influenza A isolates	73	35	136	202
(Number subtyped)	(10)	(20)	(0)	(159)
Influenza B isolates	0	9	1	289
Providers submitting one or more cultures	25	34	196	327

^a^The influenza season was defined as the period beginning with week 40 of the first year extending through week 20 of the next year, i.e. early-October though mid-May. ^b^Rapid tests incorporated into influenza surveillance during this season.

A total of 1,015 (91%) and 2,101 (97%) of the culture specimens received at HPHVL were processed through one of the participating private laboratories during the 1999-2000 and 2000-01 influenza seasons, respectively; 2,979 (96%) of these culture specimens had a rapid test performed on an accompanying specimen; the remaining specimens were direct submissions to HPHVL. All four of the private laboratories materially participated in the surveillance activity; the mean number of specimens submitted by participating laboratories was 237, with totals ranging from 188 to 333 during the 1999-2000 influenza season.

None of 136 influenza A isolates obtained during the 1999-2000 influenza season were subtyped at HPHVL. However, 159 (79%) of 202 influenza A isolates identified during the 2000-01 were subtyped and characterized at HPHVL; 143 were A/New Caledonia/20/99-like (H1N1), and 16 were A/Panama/2007/99-like (H3N2).

### Influenza Cultures submitted to WHO collaborating Laboratories in the United States

A 5% increase was observed in the number of influenza cultures submitted between the 1998-99 and 1999-2000 influenza seasons for the 37 WHO comparison sites overall (Table 2). When data for each laboratory were examined individually, Hawaii HPHVL demonstrated the largest rise in influenza cultures processed between the 1998-99 and 1999-2000 seasons (181%); the next highest increase was reported from a laboratory in the Mountain region, whose specimens increased from 260 to 524 (a 102% increase) between the 1998-99 and 1999-2000 seasons (individual data for laboratories other than Hawaii are not shown). Three other laboratories had increases of approximately 50% over the previous influenza season, but more than half (19 of 37) of the laboratories had fewer specimens submitted in the 1999-2000 season when compared to the previous season.

**Table 2:** Number of influenza cultures performed at 37 World Health Organization collaborating virology laboratories located throughout the United States (excluding Hawaii), aggregated by region and compiled by influenza season

	Influenza season			
	1997-1998	1998-1999	1999-2000	Change from 1998-1999 to 1999-2000
Hawaii	306	396	1,112	181%
Regional U.S. totals				
East South Central	190	339	424	25%
West North Central	7,225	7,218	8,663	20%
Mountain	4,456	5,531	6,471	17%
Mid-Atlantic	3,421	3,785	4,191	11%
Pacific (excl. Hawaii)	2,288	3,214	3,353	4%
South Atlantic	8,510	8,637	8,368	-3%
West South Central	1,040	2,291	2,173	-5%
New England	1,308	1,720	1,555	-10%
East North Central	3,917	4,041	3,572	-12%
Total for laboratories outside Hawaii	32,355	36,776	38,770	5%

^a^ Data for the 2000-2001 season were not yet available at the time this report was compiled.

### Physician Visits for Influenza-Like Illness

The number and proportion of patients with influenza-like illness reported from sentinel physicians in Hawaii for the past four consecutive influenza seasons are shown in Table 3. The percent of patient visits for influenza-like illness during the 1999-2000 and 2000-01 influenza seasons was generally similar to that reported during the two prior seasons. The number of weeks that this proportion exceeded baseline were 21 for the 1997-98 and 1998-99 influenza seasons and 17 for the 1999-2000 and 2000-01 seasons.

**Table 3:** The number and proportion of visits for influenza-like illness (ILI) reported from Hawaii physicians participating in the U.S. Influenza Sentinel Physician Surveillance Network, by influenza season

Influenza season	Number of participating physicians	Number of visits for ILI ^a^	Total number of visits	Overall % of visits for ILI	Number of weeks proportion of visits for ILI exceeded baseline ^b^	Highest weekly proportion of visits for ILI
1997-1998	11	805	25,085	3.2%	13	10%
1998-1999	11	728	31,308	2.3%	8	6%
1999-2000	7	272	14,831	1.8%	6	8%
2000-2001^c^	15	819	30,722	2.7%	11	6%

^a^ILI is defined as a fever of > 37.8° C accompanied by a cough or sore throat.  ^b^ILI baseline is defined as < 3% (see reference 10). ^c^Data avaialble through May 16, 2001.

### Comparing the Influenza Rapid Test with Culture

A two-by-two table comparing the results of the Flu OIA test and culture results obtained during the 2000-01 influenza season is presented (Figure). In our setting, the positive predictive value of the Flu OIA was 51%, and the negative predictive value was 84%.

**Figure:**
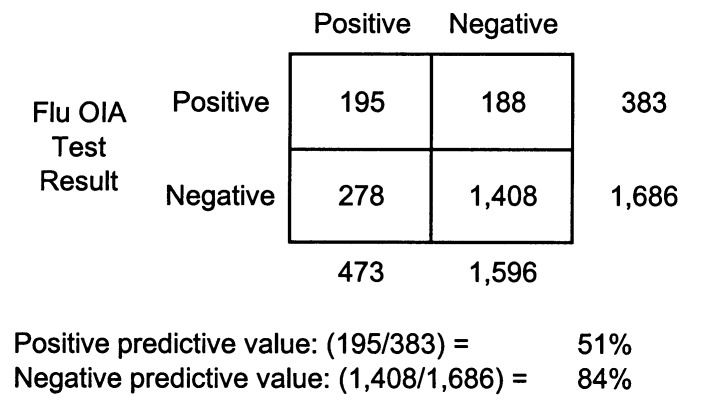
Comparison of FLU OIA and viral culture results, 2000-01 influenza season, Hawaii.

## Conclusion

This is the first evaluation of the effects of integrating rapid testing for influenza into public health surveillance. Surveillance incorporating rapid tests greatly increased the number of specimens submitted for viral culture and the number of influenza isolates obtained. This finding is important because enhancing our capacity to recover circulating influenza virus isolates is the first step in establishing a robust virologic surveillance system designed to detect novel viral strains with pandemic potential.

Although physicians were permitted to send specimens directly to HDOH for viral culture, most of the influenza culture specimens submitted during the 1999-2000 and 2000-01 seasons were collected concurrently with a specimen for rapid testing. The fact that so few culture specimens were collected without a companion specimen for rapid testing implies that many physicians felt there was value to having both a culture and rapid test result, or alternatively, that the rapid test served as an incentive to obtain a culture.

The dramatic increase in the number of physicians who participated in the enhanced influenza surveillance project as compared to standard virologic surveillance suggests that the availability of the rapid test may have appealed to a larger cohort of clinicians. The involvement of a greater number of physicians is a promising development because public health officials may ultimately be able to obtain a more extensive and representative sample of circulating influenza isolates by broadening the pool of patients sampled.

This assessment is limited in that it lacks a control group consisting of physicians and laboratories that did not have access to the free rapid influenza tests for comparison once the enhanced virologic influenza project was initiated, beginning with the 1999-2000 influenza season. Instead, we compared the number of specimens received during the 1999-2000 and 2000-01 influenza seasons to the number received in the two prior seasons. Because the severity of annual influenza seasons can vary greatly from year to year, it could be argued that the increased number of specimens submitted during the latter seasons was secondary to increased numbers of influenza-like illnesses during those seasons. Several sources of information suggest this was not the case, however. First, influenza-like illness surveillance in Hawaii over the 4-year period does not suggest that influenza activity was markedly increased during the last two seasons. Second, the number of influenza cultures performed at laboratories on the mainland during the 1999-2000 season did not increase over the previous season to the extent observed in Hawaii. Third, CDC’s published assessment of the 1999-2000 influenza season was that the “season’s activity was similar to the previous two” and influenza activity during the 2000-01 season was assessed as “moderate and lower than the previous three seasons” (*12*,*13*).

During the first season in which rapid tests were introduced, HDOH was unprepared for the increased numbers of submissions for influenza culture. HPHVL reported that the high volume of specimens received during the peak period placed an unexpected strain on virology section’s resources and that shortages in host cells, incubator space, and laboratory supplies resulted in a delay of several weeks for processing approximately 100 specimens (S. Naka, pers. comm.). The increased workload from processing specimens at HPHVL greatly reduced the laboratory’s resources for subtyping the influenza A isolates obtained that season. While recovering isolates is a critical first step in developing our capacity for influenza surveillance, to truly improve pandemic preparedness any increases in virus isolation must be coupled with expanded subtyping of influenza A isolates. Recognizing this, and with the experience gained from the prior year, adjustments in staffing and procurement permitted 79% of the influenza A isolates to be subtyped during the 2000-01 season.

Because of the difficulties encountered in processing specimens during the 1999-2000 influenza season, we limited our assessment of the influenza rapid test as compared to culture to specimens received in the 2000-01 season. This project was not designed as an evaluation of the rapid influenza test: We did not control for patient selection, specimen collection, or laboratory technique. Therefore, the findings from this field evaluation may not represent the rapid test’s performance in other settings. Other assessments on the performance of influenza rapid tests are available (*5*,*7*,*14*).

Finding that isolates recovered during the influenza season increased when we integrated influenza rapid testing into our surveillance system should help allay concerns that adopting these tests will undermine virologic surveillance. If rapid influenza tests become an established component of clinical management, it will be difficult to convince clinicians to collect culture specimens in lieu of performing rapid tests (*7*,*15*). Collection of a second specimen for culture confirmation, on the other hand, may be seen as clinically appropriate. Therefore, public health agencies may ultimately benefit from incorporating rapid diagnostic tests into influenza surveillance programs.

None of the commercial laboratories that participated in this project were independently offering rapid influenza testing before the 1999-2000 season. Through this initiative, each of the laboratories gained a level of proficiency in performing the rapid influenza test, and physicians became familiar with ordering the test. Once rapid influenza testing becomes established in laboratory and clinical practice, the question of whether providers will continue to opt for a rapid test and viral culture provided by a public health agency, or simply order a rapid test from a private laboratory at the patient’s expense, is still unanswered.

Although Hawaii’s Enhanced Influenza Virologic Surveillance program is a good model of a public-private partnership (*16*), we have one potential concern about the impact of using rapid influenza tests on public health surveillance. To date, at least two private influenza surveillance systems using rapid tests have been established--one by a manufacturer of a rapid test kit and the other by a pharmaceutical company that produces an anti-influenza medication (*17*,*18*). Large private disease surveillance systems are relatively new to medicine. Because the chief concern of these proprietary systems is likely to increase sales of a product, it is not clear what role they will play in protecting the public health. If private surveillance systems compete with public agencies for physician participation, they may adversely affect virologic influenza surveillance.

In summary, our findings demonstrate that rapid influenza tests can be successfully integrated into public health surveillance efforts, resulting in a larger number of influenza isolates being available for subtyping and antigenic characterization. This enhanced influenza surveillance effort was accomplished through mutually beneficial public-private partnerships with commercial laboratories that routinely provide service to community physicians and their patients. Preparing for the next influenza pandemic compels public health agencies to work with physicians to expand our capacity for influenza surveillance. As the available data indicate that recent influenza pandemic strains have originated in Asia, vigilant virologic surveillance is especially important for Hawaii (*19*). Should an anomalous influenza strain emerge from Asia again in the future, our state’s unique geographic location and visitor profile make it likely that our population will be among the first in the United States to encounter this new pathogen.
